# Public Awareness and Perceptions of Invasive Alien Species in Small Towns

**DOI:** 10.3390/biology10121322

**Published:** 2021-12-14

**Authors:** Nolwethu Jubase, Ross T. Shackleton, John Measey

**Affiliations:** 1 Kirstenbosch Research Centre, South African National Biodiversity Institute, Cape Town 7405, South Africa; 2 Centre for Invasion Biology, Department of Botany & Zoology, Stellenbosch University, Stellenbosch 7599, South Africa; ross.shackleton@wsl.ch (R.T.S.); jmeasey@sun.ac.za (J.M.); 3 Institute of Geography and Sustainability, University of Lausanne, 1015 Lausanne, Switzerland; 4 Swiss Federal Institute for Forest, Snow and Landscape Research, WSL, Zürcherstrasse 111, 8903 Zürich, Switzerland

**Keywords:** biological invasions, environmental education, public perceptions, management, stakeholders

## Abstract

**Simple Summary:**

Every year, the South African government spends approximately ZAR 2 billion to control invasive alien species (IAS) that are intentionally or unintentionally introduced into new areas by humans, and have a variety of social, ecological, and economic impacts. Given the link between people and the introduction and spread of IAS, it is important to understand citizens’ knowledge and perceptions of IAS to guide management. For this reason, we conducted a study in small towns of South Africa to assess (1) awareness of IAS by the general public, (2) local perceptions of the impacts associated with IAS, (3) if awareness of IAS is associated with demographic covariates and IAS density, and, (4) people’s willingness to detect, report, and support IAS management. We found that people were not aware of IAS and their impacts, and many perceived them as beneficial. We found that IAS density, education level, and gender influenced people’s knowledge and perceptions about IAS in the region. Some people showed interest and willingness to learn more about IAS. These results could help to inform outreach and educational programs to promote public awareness and engagement in IAS management.

**Abstract:**

Invasive alien species (IAS) are a growing threat globally and cause a variety of ecological, economic, and social impacts. People can introduce IAS and facilitate their spread, and can also implement, support, or oppose their management. Understanding local knowledge, awareness, and perceptions are therefore crucial if management and policy are to be effective. We administered questionnaires to members of the public in eight small towns along the Berg River Catchment in the biodiverse fynbos biome of South Africa. We aimed to assess: (1) awareness of IAS by the general public, (2) local perceptions of the impacts associated with IAS, (3) whether awareness of IAS is correlated with demographic covariates and IAS density, and (4) people’s willingness to detect, report, and support IAS management. Overall, 262 respondents participated in the survey. Most respondents (65%) did not know what IAS are, and 10% were unsure. Many respondents also perceived IAS as beneficial. Using a logistic regression, we found that IAS density, educational level, and gender influenced people’s knowledge and perceptions about IAS in the region. There were a small number (4%) of respondents currently detecting and reporting IAS, but many respondents were interested to learn more. We concluded that people living in small towns in the Western Cape of South Africa remain largely unaware of IAS and their impacts. It is crucial to increase awareness-raising initiatives, and build support and engagement in management of IAS in small towns.

## 1. Introduction

Invasive alien species (IAS) are a major and growing threat, and cause different ecological, economic, and social impacts around the world [1,2]. The increased movement of people and goods globally is accelerating the problem [3]. Due to people’s role in introducing and spreading IAS, there is a great need to involve and engage the public in their management [4,5,6,7,8]. There is a growing body of research on people’s knowledge and perceptions of IAS and their management, including in South Africa [9,10,11,12,13,14,15,16,17,18]. However, there are still gaps to be addressed with regards to assessing and understanding local knowledge and perceptions regarding IAS. These particularly relate to research focusing on urban areas and small towns and comparing invasion densities with peoples’ knowledge and perceptions. While cities are hotspots for invasions [19,20], small towns have a greater chance of facilitating invasions into natural areas [21,22], and are therefore a potential gateway to major invasions. Despite the threat rural areas pose for biological invasions, we expect that people living in small towns are probably still unaware of IAS, their negative impacts, and the role they can potentially play in preventing their spread or controlling them, as there is less engagement and awareness raising initiatives in these areas [23,24].

Several actions are considered important in the prevention and management of biological invasions, most of which rely on educating the public about IAS issues [7,25]. Improved education on IAS could help prevent IAS spread and promote control. People transport IAS to new areas and therefore serve as the primary pathway of spread. Changing stakeholder perceptions, behaviours, and attitudes about IAS can also increase support for management [26,27,28,29,30]. In the UK, for example, awareness raising and behaviour change surrounding the spread of aquatic IAS was promoted through the fairly successful “check, clean, dry” campaign [31]. The public can also play an important role in broad-scale surveillance and monitoring programmes that can aid management [18,32]. If IAS are perceived as beneficial, the public can also obstruct management interventions. Therefore, understanding people’s awareness and perceptions of IAS can be useful to guide future awareness activities, management, and research.

Despite the major impacts of biological invasions in South Africa [33,34]; the high profile research, legislation, and management in the country [35,36]; and numerous and diverse attempts at raising public awareness of IAS [24,37], existing research suggests that people are relatively unaware of IAS, especially in urban areas [13,23,38]. This low awareness could be due to many reasons, such as urban residents facing limited exposure to IAS and their impacts [23,39,40], or limited engagement with environmental systems. The limited awareness of biological invasions may hinder IAS control, resulting in their increased spread and associated negative impacts, as well as potentially leading to greater conflicts over management [41,42]. According to [43], increased awareness raising and detection efforts should initially target populated areas, because most plants first naturalise in urban areas due to the high concentrations of gardens where ornamental alien species are introduced. Research by [44] supported this view, and showed the potential for gardens in small towns in South Africa as launching sites for many alien plant invasions. For example, [44] recorded 298 alien plant species in the small town of Riebeek Kasteel that were either naturalised or invasive in the Berg River catchment in the Western Cape, South Africa. Most of these alien plant species occurred in peoples’ gardens and along roadsides.

In this study, we built on the work of [44] by administering questionnaires to members of the public in eight small towns along the Berg River Catchment, which is situated in in the biodiverse fynbos biome of South Africa. The study aimed to assess: (1) awareness of IAS by the public, (2) public perceptions of the impacts associated with IAS, (3) whether awareness of invasive species is correlated with demographic covariates and IAS density, and (4) people’s willingness to detect, report, and support IAS management.

## 2. Methods

### 2.1. Study Site

**Figure 1:**
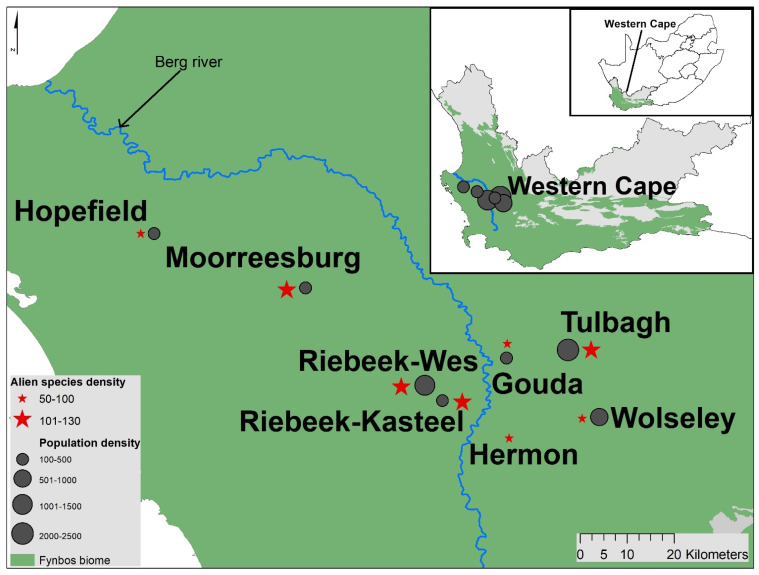
Location of the Berg River catchment in South Africa’s Western Cape Province and eight small towns selected for this study, with population densities and alien species density in each town. Population density data for Hermon was not available. Estimates of population density and alien density are from McLean et al. [21].

The Berg River Catchment in the Western Cape of South Africa has 28 towns with detailed alien plant occurrence data [21] (Figure 1). For this study, we selected eight towns across the catchment, with different population sizes and alien species threat levels [21] for comparison. The Western Cape provincial government has substantial interest in environmental issues in the area, including the management of IAS [45,46], as this is a key water-catchment area for agriculture and the city of Cape Town. Urban areas in the Berg River Catchment range in population size from 330 to just over 100,000, with population densities ranging from 10 to 5000 people/km^2^. The area supports mainly dryland agriculture (primarily wheat). This has led to large-scale transformation of historic natural landscapes. In these areas, many of the plants remaining in seminatural fragments are IAS that have been listed in South African legislation: the National Environmental Management: Biodiversity Act ((NEM: BA), Act 10 of 2004), along with fynbos shrublands with high species diversity. The area is dominated by fynbos biome, which is the most invaded biome in South Africa, and where research and management of IAS is mostly conducted [35,47]. The management of IAS in this area is principally conducted by the Working for Water (WfW) programme launched by the South African government in 1995 [48].

### 2.2. Questionnaires/Face-to-Face Surveys

Questionnaires were conducted with 30–40 randomly selected people (>18 years of age) in each of the eight towns except for Hopefield, where only 10 questionnaires were conducted. People were directly approached and asked if they were willing to participate in the survey. We targeted people in their leisure time when they were willing to take their time to participate in the survey. We conducted face-to-face surveys between February 2018 and November 2019 during the week (between Monday to Friday) from 10h00 to 16h00, using a translator when necessary. Interviews were conducted in English, isiXhosa, and Afrikaans. In each town, we visited both administrative town centres and townships. In South Africa, a township is a residential area that was previously designated for Black residents during the Apartheid regime.

Questionnaires consisted of five sections relating to: (1) respondents general awareness about IAS, (2) their ability to identify species that were illustrated with pictures, (3) their perceptions on the benefits and negative impacts of these target species and IAS in general, (4) their attitudes towards the detection and management of IAS, and (5) socio-demographic information, which included information such as age, gender, level of education, and residence time in the town (see Supplementary Material S1). The questionnaire contained both closed-ended and open-ended questions, with an average completion time of 15 min.

### 2.3. Species Selected

To better understand people’s awareness of IAS, we selected three invasive plant species, two invasive animal species, and one indigenous species as a control, and asked people if they could identify each species from a series of photographs. All selected plant and animal species have been recorded as present within the studied area [21,49,50]. Plants that were chosen represented different growth forms (tree, grass, and herb/shrub), and examples of vertebrate and invertebrate animals were selected. Our key criterion in species selection was that it should be present in each town (using data from [21]), and therefore that our respondents had likely encountered the IAS before we conducted the survey. The specific IAS selected were: *Metrosideros excelsa* Sol. ex Gaertn (New Zealand Christmas tree), *Genista monspessulana* (L.) L.A.S.Johnson (French Broom), *Pennisetum setaceum* (Forssk.) Chiov. (Fountain Grass), *Harmonia axyridis* Pallas (Asian Ladybird), and *Sus scrofa* Linnaeus (Feral Pig). The indigenous plant species selected was *Aloe arborescens* Mill. (Krantz Aloe). The pictures for each selected species were embedded in the questionnaire, and another separate document with higher-quality pictures were shown to participants. A short description for the selected species is provided in Supplementary Material S2.

### 2.4. Analysis

In addition to the descriptive statistics, we also used R (v3.6.3) to run generalised linear models (logistic regression) to assess if awareness of IAS was correlated with invasion density (how much people knew in relation to the number of IAS in their towns) and demographic variables. Thus, the dependent variable (categorical) was the binary response from respondents stating whether or not they knew the meaning of IAS, and the independent variables were gender (categorical), age (categorical: 18–29, 30–39, 40–49, 50–59, 60–69, >70), educational level (categorical: Bachelor’s degree, Honours degree, National Diploma, Matric, High School, Primary, and N/A), and alien density (continuous). For the analysis, respondents that were unsure if they knew what an IAS was were included in the “no” response category. If they were not sure but defined the term IAS correctly, they were moved to the “yes” response category. Location was not used in the analysis, as alien density was coded through location information in each town (i.e., they covaried). Collinearity of variables was tested.

Further, we assessed if recognition of the selected plant species (*M. excelsa* and *P. setaceum*) was correlated with this species’ density in each town. We used these two species because species density data was available for them [21]. In this regard, the recognition of the species (categorical: yes/no) was used as the dependent variable, and species density as the independent variable (continuous). Species density data (coded as a continuous variable per town) were taken from McLean et al. [21]. Candidate linear models were built from each of the explanatory variables, which combined when they demonstrated significant interactions. An additional null model was included with candidate models for selection. To select the best model, we used Akaike’s information criterion (AIC) [51] to compare candidate models (see Supplementary Material S3 Tables S1 and S2). Models within 2 δAIC were considered equal. See Supplementary Material S4—for the summary of the predictor variables used, their explanation and variable types.

### 2.5. Ethics

Ethical clearance to conduct the research was obtained from the Research Ethics Committee (REC): Humanities at Stellenbosch University—Project number: 2019-9578. All ethical standards were adhered to. Informed consent was obtained from each participant, and anonymity was assured.

## 3. Results

### 3.1. Demographic Characteristics

In total, 262 people participated in the survey. Overall, there was a higher proportion of female respondents (58%) than males (42%), disproportionately allocated among towns (Supplementary Material S5). Respondents were primarily young to middle-aged, with low formal education levels (i.e., never finished secondary school), and long-time residents (born there, or lived in town for more than 10 years).

### 3.2. Awareness of the Term Invasive Alien Species

**Figure 2:**
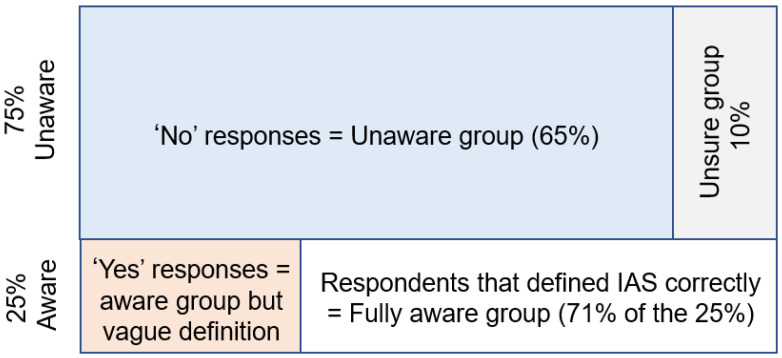
A summary of how the 262 respondents were grouped according to their level of knowledge of invasive alien species.

**Figure 3:**
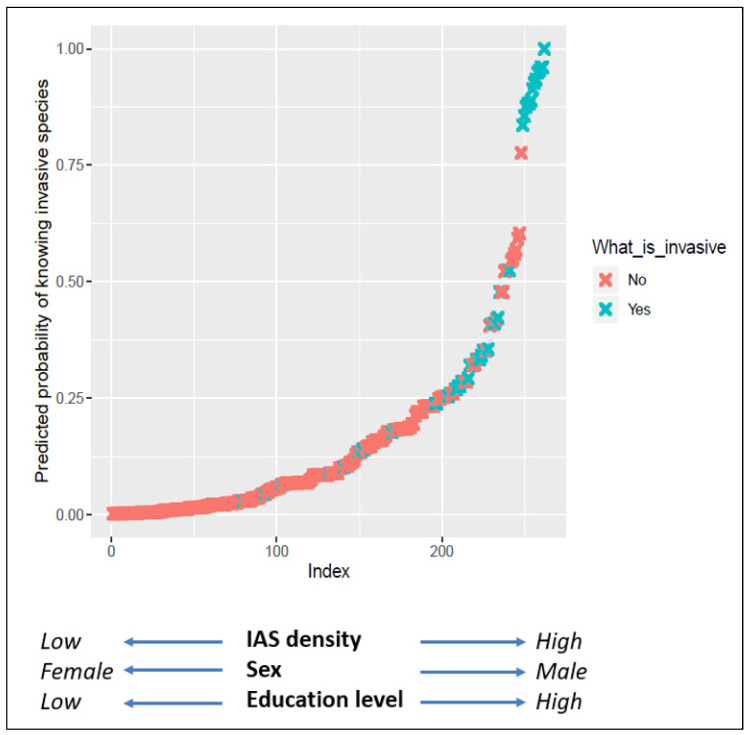
The relationship between awareness of IAS and alien plant density. The graph shows that awareness on IAS was linked to invasive plant density. The best candidate model selected had explanatory variables of higher education, higher alien density, and gender (men) to explain awareness of the term IAS by people. ‘Index’ represents the best model in Table 1.

In response to the question: “*Do you know what an invasive alien species is [yes] [no] [unsure]: if yes, describe what IAS is*”, more than half of the respondents (65%) said they did not know what an IAS was, and 10% were unsure (Figure 2). We classified these respondents as those that were “unaware”. The other “aware” group of respondents (25%) professed familiarity with the term. Of these, most (71%) were able to give a correct definition. Those that were able to give a definition that aligned with aspects of the one of the many scientific definitions were classified as the “fully aware group”. For example, a notable number of respondents defined IAS along the lines of: ‘plants that do not belong in our country that may cause harm to the environment and human life, and use up a lot of water’. This closely followed the narrative provided by the Working for Water program. The results from the logistic regression showed that alien species density, educational level, and gender influenced people’s knowledge and perceptions about IAS in the region (Table 1; Figure 3). The correlation between knowledge of IAS and each demographic variable was significant except for age (Supplementary Material S6).

We also asked all groups (aware, unaware and fully aware groups): “*Do you know what an indigenous species is?*” Most respondents (73%) did not know the term, while others were unsure (6%). Only 21% of all respondents said they knew what an indigenous species was, with 6% of the 21% giving an incorrect definition.

We further asked all groups: *“How would you categorise your knowledge of invasive and indigenous flora“? [very good] [good] [average] [very poor] [I don’t know]*. Most of the fully aware respondents (*n* = 47) considered that they had only average awareness of IAS (49%), while a minority of them thought that their awareness was good (11%) or very good (4%). Some of the fully aware respondents categorised their own level of awareness of IAS as very poor 6% or poor (26%), and (4%) of the respondents did not know.

### 3.3. Awareness of Invasive Alien Species in the Region

All groups of respondents were asked: *“Can you name any IAS occurring in your town or in South Africa”? [yes] [no] [unsure]*, and if yes, we listed the species they mentioned. Those who could define IAS were more likely to correctly name one. Seventeen species were specifically mentioned by respondents as IAS occurring in their small towns and in South Africa. *Aloe arborescens* Mill. was mentioned as alien by two respondents; it is native to South Africa, but not indigenous to the Berg River Catchment. The most named IAS were *Acacia saligna* (Labill.) H.L. Wendl. (Port Jackson) (*n* = 24), *Eucalyptus* spp. (Blue Gum) (*n* = 14), *Acacia mearnsii* De Wild. (Black Wattle) (*n* = 13), and to a lesser extent *Eucalyptus grandis* W. Hill ex Maiden (Rose Gum) (*n* = 3) and *Acacia cyclops* A. Cunn. ex G. Don A. (Rooikrans) (*n* = 3). The species that were mentioned by one or two respondents included: *Lantana camara* L. (Lantana), *Melaleuca* spp. (Bottle Brush), *Pinus* species (Pine trees), *Quercus robur* L. (Oak trees), *Parthenium hysterophorus* L. (Parthenium/Famine Weed), *Acacia longifolia* (Andrews) Willd. (Long leaf wattle), *Nerium oleander* L. (Oleander), *Jacaranda mimosifolia* D. Don (Blue Jacaranda), *Greviella striata* R.Br. (Beefwood), *Gilia tricolor* Benth. (Birds’ eye), and *Schinus mole* L. (Pepper tree).

### 3.4. Awareness and Recognition of the Target Selected IAS and Whether They Are Invasive/Indigenous

**Figure 4:**
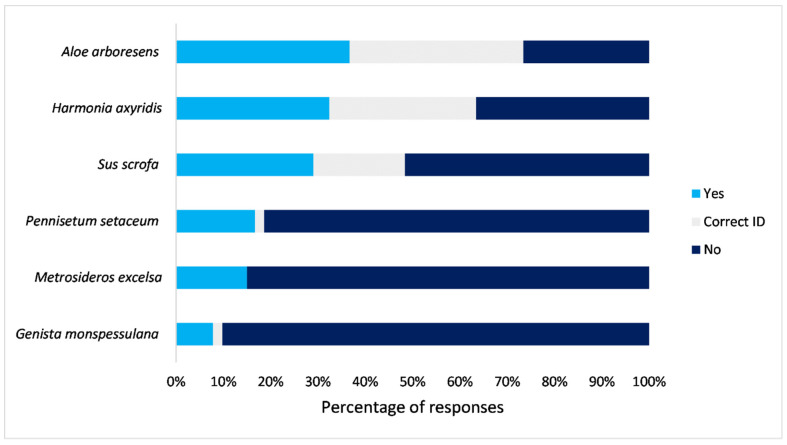
Percentage of questionnaire participants that could recognise the target species *(Aloe arborescens, Genista monspessulana, Harmonia axyridis, Metrosideros excelsa*, *Pennisetum setaceum,* and *Sus scrofa*). Light blue indicates respondents said that they could recognise the species but could not name it; grey indicates respondents that could recognise and correctly name/identify the species, and dark blue indicates those that did not recognise the species. *Aloe arborescens* is indigenous to South Africa, and the rest of the species are invasive.

**Figure 5:**
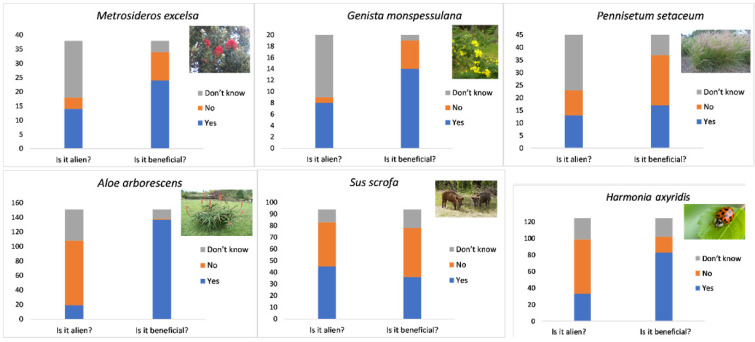
Number of respondents who knew the species in question were IAS or indigenous and the number who perceived each as beneficial or harmful. *Aloe arborescens* is indigenous to South Africa, and the rest of the species are invasive alien species found in the study region. Photo references—top row from left to right: *Metrosideros excelsa* Photo credit: South African National Biodiversity Institute, 2012; *Genista monspessulana* Photo credit: South African National Biodiversity Institute, 2010; *Pennisetum setaceum* Photo credit: Pinterest, accessed date: 30 November 2021, from: https://www.pinterest.com.mx/adrian_leon_v/pennisetum-setaceum; *Aloe arborescens* Photo credit: Plant Info, accessed date: 30 November 2021, from: https://plantinfo.co.za/plant/aloe-arborescens; *Sus scrofa* Photo reference: Thai National Parks, accessed date: 30 November 2021, from: https://www.thainationalparks.com/species/wild-boar; *Harmonia axyridis* Photo reference: Pintrest, accessed: 30 November 2021, from https://za.pinterest.com/pin/821414419519860067.

We asked all groups of respondents if they recognised the species in question (through photographs) and if they could identify them. The most correctly identified IAS were animals: *H. axyridis* (47%) and *S. scrofa* (36%) (Figure 4), although most respondents incorrectly categorised them as indigenous. This was followed by 8% of respondents who said they could identify *G. monspessulana,* with only 2% positive identifications. *Pennisetum setaceum* was recognised by 17% and correctly identified by 1% of respondents. *Pennisetum setaceum* was misidentified for *Cortaderia* *selloana* (Pampas Grass) and *Alopecurus* species (Foxtail Grass) by some of the respondents. None of the respondents could positively identify *M. excelsa,* even though 15% of respondents initially said they could recognise the species, which was mostly misidentified for *Melaleuca* sp. (Bottlebrushes). The correct species identifications were irrespective of whether respondents knew if the plants or animals in question were indigenous or invasive. Knowledge of whether the species under question were invasive or indigenous varied between species (Figure 5). Just over half (51%) (Figure 5) of respondents did not know the status (whether they were invasive/indigenous) of the selected target species.

Most respondents from all groups (58%) were able to recognise and identify *A. arborescens* correctly, and some of these respondents (59%) knew that the species was indigenous to South Africa. Only 13% of these respondents classified it as IAS, while other respondents (28%) said they did not know whether the species was invasive or indigenous, even though they knew the plant.

The best linear model explaining recognition of two invasive plant species (*M. excelsa* and *P. setaceum*) related to the relative higher density of these species in different towns in both cases. Specifically, the best model explaining identification of *M. excelsa* by people included higher density of *M. excelsa* and higher education level (*p* = 0.0019). This was equivalent to 10.46% of the variation in the data. The best model explaining the recognition of *P. setaceum*, related to people with higher education levels, *P. setaceum* density, and respondents age (younger respondents aged 18 to 29 were better able to identify the IAS). This was equivalent to 16.49% of the variation in the data, and was significant (*p* = 0.0001; see Supplementary S3 Tables S1 and S2 for all candidate models).

### 3.5. Willingness to Detect and Report IAS

There were a small number (*n* = 10; 4%) of respondents currently detecting and reporting IAS around 1–5 times a year. These respondents reported their sightings mainly to project managers at CapeNature and Working for Water, and seven of the 10 respondents currently also worked for these organisations.

We asked all respondents, “*Would you be interested in learning how to report the localities of IAS?”.* Most respondents (56%) did not show any interest in learning about detecting and reporting IAS, whereas 44% showed interest regardless of whether they were aware or unaware of what an IAS was before the survey. Generally, people showed interest to broaden their knowledge around the issue of IAS. For example, one respondent said, “*maybe these species can be harmful, and I need to know where to report them and find help*” while another respondent said, “*I want to report all the Port Jackson on my neighbour’s property*”. Other reasons for wanting to learn were associated with negative impacts of IAS. For example, “*it’s good to know more about invasive species and identify them because they take up a lot of water*”. The reasons for not wanting to learn more about detecting and reporting IAS varied amongst respondents. For example, one respondent said, “*Leave plants alone, they give us oxygen*”. Other respondents said they do not have time or were too old to learn about them: “*I am too old now to learn about these things*”.

### 3.6. Willingness to Support IAS Management

We asked all respondents, *“Would you like to see IAS removed and densities decreased in your area”? [yes] [no] [unsure] [I don’t care].* In response to this question, 44% of respondents were unsure if management of IAS was necessary (7% were from the aware group and 3% were from the fully aware group). A minority of respondents from all three groups (21%) were completely against the removal of IAS. Of these respondents, 8% were from the aware group and 19% were from the fully aware group. Some respondents said, “*Plants are good for the environment and they bring more oxygen*” and “*they have to be replaced with other trees because they create oxygen*”, while other reasons for not wanting management related to aesthetic and utilitarian benefits of IAS; for example, one respondent said, “*Its nature, plants are beautiful, why remove them?*”

Almost a third of respondents (28%) from all groups agreed that management of IAS is necessary due to negative impacts that these species cause. Most (55%) of respondents that agreed that management of IAS is necessary were from the aware and fully aware group (44% of respondents from the fully aware group and 11% respondents from the aware group). Some respondents did not care (7%, with 12% of these respondents from the aware group and another 12% from the fully aware group). Reasons for not caring mainly revolved around views of limited impacts; for example, one respondent said, “*It does not affect me, and I don’t have knowledge of IAS causing trouble in our town at the moment*”. Respondents who had prior experience and awareness of IAS supported their control more than those who did not have the prior awareness (Table 1).

Participants from all groups were asked, *“Have you ever volunteered to manage IAS?” [yes] [no]* and *“Would you like to join groups that manage/control IAS in your area”? [yes] [no]*. Voluntary participation in IAS management was low among all groups of respondents. Most respondents (89%, 18% from the aware group and fully aware group) said they had never removed IAS, while a few respondents (11%) had been involved in IAS management programmes before, whether they did it in their homes or at work.

Only 6% of respondents (of which 19% were from the aware group and 50% from the fully aware group) were associated with volunteer groups managing IAS before. Only 10% of respondents from all three groups said they would be happy to join local volunteer groups that manage IAS; 19% of these respondents were from the aware group, and 54% were from the fully aware group.

### 3.7. Willingness to Learn More about the General Issue of IAS

Respondents were also asked, “*Would you like to learn more about Invasive species and their impacts”? [yes) [no] [unsure].* Just over half of respondents (53%) (63% from the unaware group and 37% from the aware and fully aware group) were interested and willing to learn more about IAS and their impacts, whereas 16% said they were unsure (91% from unaware group and 9% from the aware and fully aware group). Almost a third of respondents said they were not interested in learning more (31%) (87% from the unaware group and 13% from the aware and fully aware groups). No reasons were given by respondents as to why they said that they did not want to learn more about IAS. We further asked all participants if they would like to receive information on IAS, and most respondents (55%) said yes. Respondents who had previously heard of IAS and their impacts were more likely to want to learn more about IAS than those who had little or no knowledge of IAS.

We asked all respondents, *“Have you ever read any articles with information about invasive alien species? [yes] [no], if yes, where?”* Most respondents (86%) said they had never been exposed to information on IAS. Other respondents (3%) mentioned they got information and learned about IAS through their jobs at conservation, forestry, education, and local government institutions. Local newspapers (3%) and magazines (3%) were also mentioned as important mediums of communication where a few respondents had learned about IAS. A few respondents said that they learned about IAS at school (2%), and 1% of respondents said they learned about IAS from the television. A few respondents mentioned that they learned about IAS and their impacts through personal observations and experiences (2%).

## 4. Discussion

Our results demonstrated that the meaning of invasive alien species (IAS) is understood by a minority of people in small towns in the Berg River catchment of South Africa. We found that alien species density, education level, and gender influenced people’s knowledge and perceptions of IAS in the region. This was similar to the findings of previous research in which awareness and understanding of the concept of IAS was associated with people with higher education levels, especially in South Africa, where education levels differ enormously among groups of people due to the legacy of apartheid [13,23,39]. We did not come across any published information associating higher IAS awareness with gender. In our study, men had higher awareness levels, which we believe could be linked to their occupations (often more environmentally or agriculturally linked). Our findings also suggested that higher alien species densities also led to great awareness surrounding IAS. There is previous work linking IAS density, awareness, and perceptions [23], although this is not highly comprehensive.

The majority of the respondents did not know whether the species presented in images (many of them common in the research area, see [21]) were indigenous or invasive, and did not know their impacts. Even though our linear model results showed that IAS knowledge was correlated with alien species density, we found that the overall level of awareness of the target selected invasive plants and their impacts (*P. setaceum*, *G. monspessulana* and *M. excelsa*) was low, with invasive animals *H. axyridis* and *S. scrofa* being better identified. This supported the notion that people did not have a good knowledge of IAS in South Africa, even those in their own communities [23,41]. Most respondents from all three groups categorised *H. axyridis* and *S. scrofa* as indigenous even though they are IAS; there are, however, similar looking native species in South Africa. In addition, *H. axyridis* was correctly identified by 47% of respondents but perceived as beneficial, which suggested possible low support for the management action of these invertebrates. Many respondents perceived IAS as beneficial (Figure 5), especially for aesthetics, which showed that IAS can have important value to people regardless of them being invasive or not [42,52]. Our results echoed those of [38], in that the species’ taxonomic position (i.e., animal or plant) may influence public knowledge and support for the management action [17,38].

Surprisingly, many respondents did not know the term indigenous either (this is the commonly used term in South Africa). Despite Western Cape being the most invaded province in South Africa and a global biodiversity hotspot [35], the public from the eight small towns remained largely unaware of not only IAS, but also of other related biological terms. This supported the view that fundamental biological concepts were historically not being communicated and taught to large groups of people in South Africa. It was only in 2007 when both of these terms were included in the national school curriculum [24]. For younger respondents, it might be possible that the concepts were communicated but this information was not retained. Issues with communicating fundamental biological concepts has also been recognized in South Africa and other regions of the world. For example, [39] concluded that the public in California, USA had poor understanding of IAS. However, Californians were more familiar with the term “weed”, which was understood to be more of a nuisance than an environmental problem. Similarly, [53] discovered that people in the Canton of Zurich, Switzerland were still unfamiliar with the term biodiversity and associated concepts such as IAS. In [54], it was found that there were few landowners who knew about IAS in a West Virginia woodland in the USA. In a study in Grahamstown (Makhanda), South Africa, [23] found that less than half of the respondents could identify an invasive tree on their property. Another study by [13] highlighted factors such as how apartheid’s legacy contributes to different knowledge levels of IAS across different communities in Cape Town, South Africa. Low levels of awareness of IAS and their status as listed invasive or indigenous species, and their impacts amongst people implies that the NEM:BA invasive species regulations were not widely known by people in these small towns in South Africa. This will have negative implications for uptake of this policy, which, for example, mandates people to remove listed IAS from their properties. Similarly, in Europe, [16] found that most respondents (>50%) were not aware of the role and existence of the European Union IAS regulation 1143/2014.

There are many reasons that may be associated with poor awareness of IAS among people in small towns in South Africa. Firstly, it may be due to low levels of awareness raising and engagement activities with the public, which may be caused by a shortage of funding, or researchers’/managers’ capacity and time [7,30]. Secondly, people have not been told about IAS and their impacts [39]; in South Africa, the topic was only introduced to the school curriculum in 2007 [24]. Despite the lack of learning opportunities and exposure, often people may be willing to learn once engaged on the topic of IAS [40]. For example, it is encouraging that more than half of respondents (53%) in this study showed interest and willingness in learning more about IAS and their impacts regardless of their current level of knowledge. This suggested that awareness-raising initiatives could have the potential to improve IAS management efforts by the public. Another reason for low levels of knowledge is sometimes lack of interest or the resistance to information on biological invasions issues amongst the public, particularly as many people in urban areas perceive that IAS have no effect on their lives, and that they can be beneficial [40,52,53]. For example, a quarter of respondents (31%, *n* = 80) in this study were not interested in learning more about IAS, but no reasons were given by respondents as to why. This was not surprising, as other studies showed that the public does not care about IAS unless they are directly affected [38,40,55]. Lastly, people may not think of the net ecological impacts of invasions as bad, especially if they are not exposed to them; furthermore, many may perceive IAS as beneficial [17,52]. For example, when we asked respondents in this study if they would be willing to support management activities aimed at IAS, several respondents perceived that IAS have benefits; for example, that they bring oxygen. This statement may be linked to the publicity around the benefits of trees that have come from high-profile global campaigns [56,57].

This limited awareness may have implications for policy and management implementation [16,23,58], as it might make the public less engaged in control, and could therefore lead to the spread of IAS. Overall, there were only a few respondents who had the perception that IAS negatively impact biodiversity (Figure 5). We found that prior awareness of IAS was found to be an important factor in perceiving them as beneficial or harmful and for supporting control activities, which was similar to other studies [5,15,17]. Respondents who knew IAS were more likely to want to support control programmes and assist with detection efforts. This supported the view that the more the public is aware of IAS, the more they will support their management [11,13]. This makes awareness raising an important task for management.

Targeted and appropriate awareness-raising techniques are highly important factor affecting people’s involvement in environmental management. According to [55], the most suitable awareness-raising technique depended on how many people were required to be targeted, the geographical reach of the initiative, and the skills required (if any) to engage in the behaviour change. This suggested that awareness raising should be tailor-made for different groups of people in different social–ecological settings. We feel that emphasis should be on educating the public about the impacts of IAS on their lives, to build support for management and change opinions in favour of IAS control [38], as well as to change behaviours, such as getting people to instead plant indigenous species. The public should also be informed about the control efforts of IAS that are undertaken as one of the government’s largest public works programmes in South Africa, to illustrate the huge effort the state is investing in managing IAS issues.

## 5. Conclusions

This study showed that knowledge of IAS among people in small towns of South Africa was limited, and knowledge was influenced by alien species density, education level, and gender. Furthermore, the majority of respondents did not know if the species they were shown were indigenous or invasive, and generally perceived them as beneficial. This limited awareness may hinder support of IAS management and require targeted awareness-raising campaigns. We believe that localised education efforts (communicating the issue through emphasis on species that are locally invasive) would help to promote interest of IAS among the general public.

## Figures and Tables

**Table 1:** Candidate models explaining demographic covariates and species density (continuous independent variable). The models are ordered by their relative AIC, and the best model is highlighted in bold. *W*i (Akaike weight) is the relative support a model had from the data compared to the other models in the set. Significance levels (P) for models with categorical variables (*) are reported in Supplementary Material S6 (NS: not significant).

Model Description	Log Likelihoods	Number of Parameters	δAICs	*W*i	P
**What is invasive~Gender + Education +** **alien density**	**−82.4329**	**9**	**0**	**0.9965**	*
What is invasive~Gender + Education	−89.2397	8	11.61	0.0030	*
What is invasive~Gender + Residency + Age + Education + alien density	−80.1313	19	15.40	0.0004	*
What is invasive~Education	−94.2305	7	19.60	<0.0001	*
What is invasive~Location	−94.1041	8	21.34	<0.0001	*
What is invasive~alien density	−112.339	2	45.81	<0.0001	<0.0001
What is invasive~Gender	−118.561	2	58.26	<0.0001	0.0025
What is invasive~Residency	−115.84	5	58.81	<0.0001	*
What is invasive~1	−123.262	1	65.66	<0.0001	NS
What is invasive~Age	−121.524	7	74.18	<0.0001	NS

## Data Availability

Data available upon request from authors.

## Supplementary Materials

The following are available online at https://www.mdpi.com/article/10.3390/biology10121322/s1, S1: Questionnaire directed to peoples before awareness raising treatments. Draft versions of the questionnaire were jointly developed by the authors and pilot tested prior to the survey. We conducted face-to face surveys between 2018 and 2019, S2: A short description of the selected IAS: *Metrosideros excelsa, Genista monspessulana, Pennisetum setaceum, Harmonia axyridis* and *Sus scrofa*. Indigenous species: *Aloe arborescens*, S3: Table S1: showing different linear models tried to assess if knowledge of *Metrosideros excelsa* is correlated with plant densities in different towns. The models are ordered by their relative AIC and the best selected one is highlighted in bold. Table S2: showing different linear models tried to assess if knowledge of *Pennisetum setaceum* is correlated with plant densities in different towns. The models are ordered by their relative AIC and the best selected one is highlighted in bold, S4: Summary of the parametric variables used in the analyses performed at different scales, their questions/explanation and variable type. The original wording and questions are given in a Supplementary material file 1, S5 Demographic details for the surveyed population from eight towns along the Berg River Catchment, S6 Significance levels of candidate models with categorical variables explaining correlation between knowledge of IAS (marked with * in Table 1).
