# Supplementary material for: Public Awareness and Perceptions of Invasive Alien Species in Small Towns
Source: Biology (Basel). 2021 Dec 14;10(12):1322. doi: 10.3390/biology10121322 (PMC8698633; doi:10.3390/biology10121322)
Supplement: Supplementary file 1 [file biology-10-01322-s001.zip › biology-1481044-supplementary.pdf]

Supplementary information S1: Questionnaire directed toparticipants. Draft versions of the questionnaire were jointly developed by the authors and pilot tested prior to the survey. We conducted face-to face surveys between 2018 and 2019.

Questionnaire

1. Do you know what an invasive alien species is? [yes] [no] [unsure]: If yes, describe what is Invasive alien species
…………………………………………………………………………………………………
…………………………………………………………………………………………………
……………………………………………………………………………………….

2. Can you name any invasive alien species? [yes] [no]: If yes, list the ones they mention
…………………………………………………………………………………………………
…………………………………………………………………………………………………
....

3. Do you know what an indigenous species is? [yes] [no] [unsure]: If yes, describe what is an indigenous species
………………………………………………………………………………………………
…………………………………………………………………………………………………
………………………………………………………………………………………

4. Can you name any indigenous species? [yes] [no]: If yes list the first 5 they mention
………………………………………………………………………………………………
…………………………………………………………………………………………………
………………………………………………………………………………………..

5. Would you like to learn more about Invasive species and their impacts? [yes] [no] [unsure] [not interested]

6. How would you categorise your knowledge of invasive and indigenous flora? [very good] [good] [average] [poor] [very poor] [don’t know]

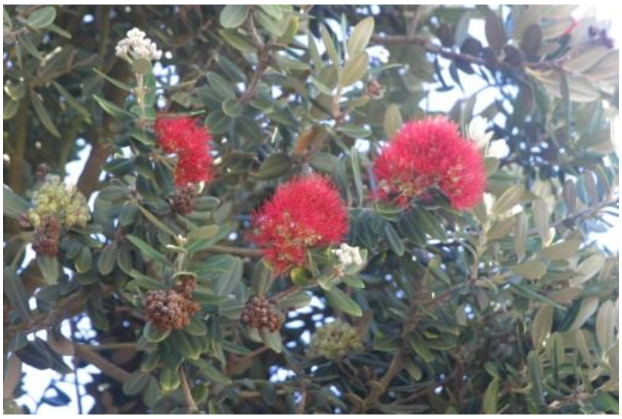 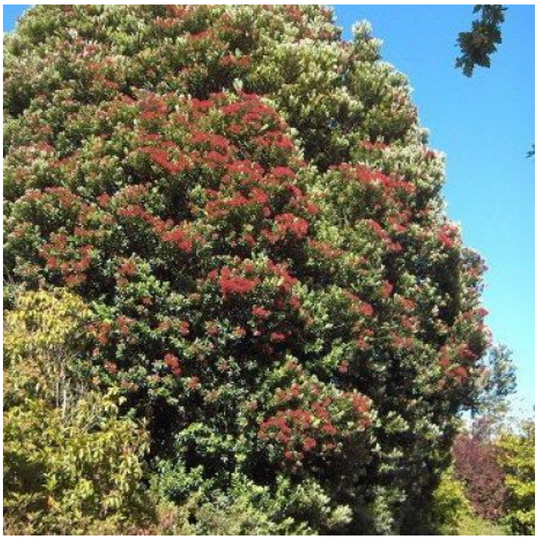

6.1. Can you identify this plant? [yes] [no]: if yes, give name of the plant ……………………………………………………………………………… and continue, if no skip rest

6.2. Is it invasive or indigenous? ……………………………………………………………………………………….....

6.3. Have you personally seen this plant? [yes] [no] [not sure]

6.4. Is it [very common] [common] [moderate] [scarce] [very scarce] in your area?

6.5. Is it spreading and increasing in your area? [yes] [no] [same] [don't know]

6.6. Is this plant [beneficial] [harmful] [no impact] or [both harmful and beneficial]? Reason for answer

………

………

6.7. Where did you learn about these benefits or impacts? [personal observation] [verbal communication] [school/university] [the media] [the internet] [other – specify]

………

………

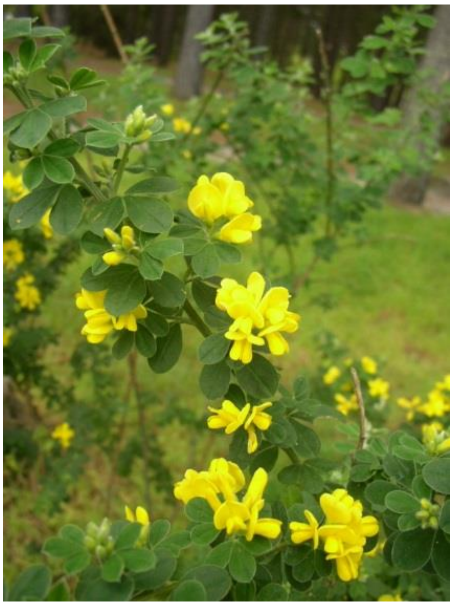
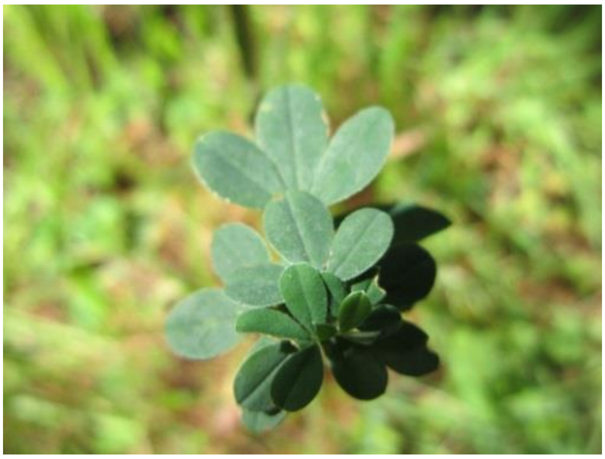

6.1. Can you identify this plant? [yes] [no]: if yes, give name of the plant ……………………………………………………………………………………… and continue, if no skip rest

6.2. Is it invasive or indigenous? ……………………………………………………………………………………………....

6.3. Have you personally seen this plant? [yes] [no] [not sure]

6.4. Is it [very common] [common] [moderate] [scarce] [very scarce] in your area?

6.5. Is it spreading and increasing in your area? [yes] [no] [same] [don't know]

6.6. Is this plant [beneficial] [harmful] [no impact] or [both harmful and beneficial]? Reason for answer

………

……

6.7. Where did you learn about these benefits or impacts? [personal observation] [verbal communication] [school/university] [the media] [the internet] [other – specify]

……

……

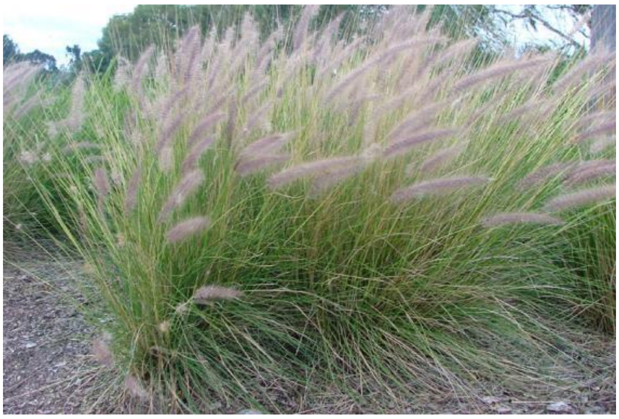 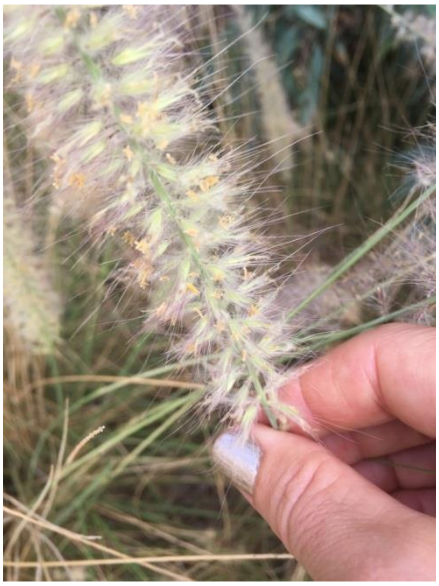

6.1. Can you identify this plant? [yes] [no]: if yes, give name of the plant ………………………………………………………………………………… and continue, if no skip rest
6.2. Is it invasive or indigenous? ………………………………………………………………………………………
6.3. Have you personally seen this plant? [yes] [no] [not sure]
6.4. Is it [very common] [common] [moderate] [scarce] [very scarce] in your area?
6.5. Is it spreading and increasing in your area? [yes] [no] [same] [don't know]
6.6. Is this plant [beneficial] [harmful] [no impact] or [both harmful and beneficial]? Reason for answer

……

……

6.7. Where did you learn about these benefits or impacts? [personal observation] [verbal communication] [school/university] [the media] [the internet] [other – specify]

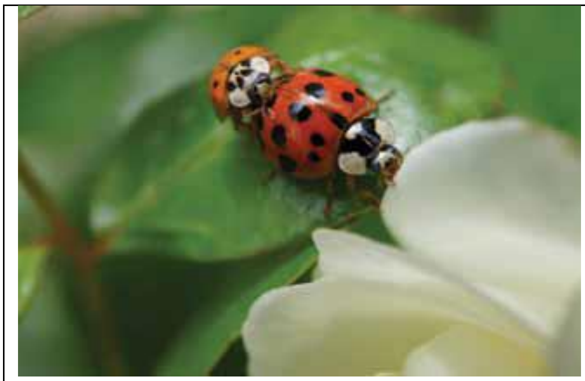 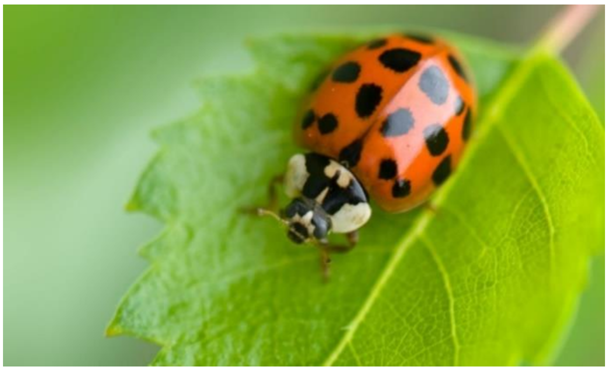

6.1. Can you identify this animal? [yes] [no]: if yes, give name of this animal ………………………………………………………………………………… and continue, if no skip rest

6.2. Is it invasive or indigenous? …………………………………………………………………………………

6.3. Have you personally seen this animal? [yes] [no] [not sure]

6.4. Is it [very common] [common] [moderate] [scarce] [very scarce] in your area?

6.5. Is it spreading and increasing in your area? [yes] [no] [same] [don't know]

6.6. Is this animal [beneficial] [harmful] [no impact] or [both harmful and beneficial]? Reason for answer

……..

……..

6.7. Where did you learn about these benefits or impacts? [personal observation] [verbal communication] [school/university] [the media] [the internet] [other – specify]

………..

……………………………………………………………………………………………………
………………………………

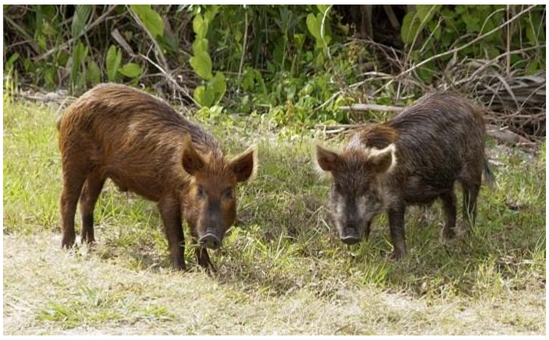
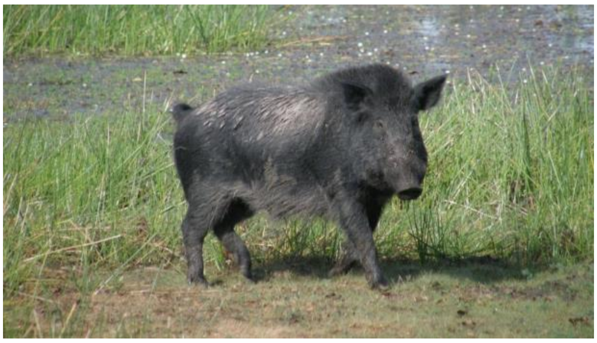

6.1. Can you identify this animal? [yes] [no]: if yes, give name of this animal …………………………………………………………………………………… and continue, if no skip rest
6.2. Is it invasive or indigenous? ……………………………………………………………………………………………...
6.3. Have you personally seen this animal? [yes] [no] [not sure]
6.4. Is it [very common] [common] [moderate] [scarce] [very scarce] in your area?
6.5. Is it spreading and increasing in your area? [yes] [no] [same] [don’t know]
6.6. Is this animal [beneficial] [harmful] [no impact] or [both harmful and beneficial]? Reason for answer

……………………………………………………………………………………………………
………………………………

……………………………………………………………………………………………………
………………………………

6.7. Where did you learn about these benefits or impacts? [personal observation] [verbal communication] [school/university] [the media] [the internet] [other – specify]

……

……

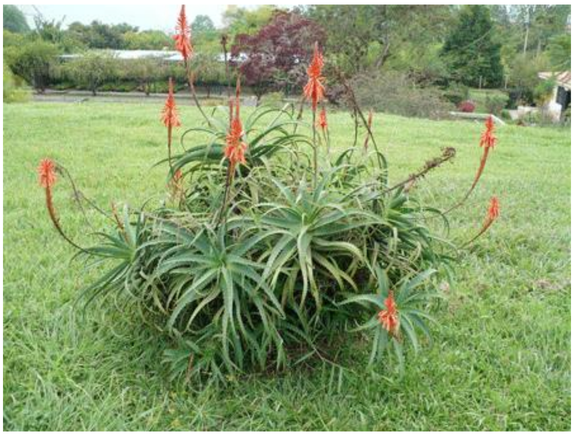

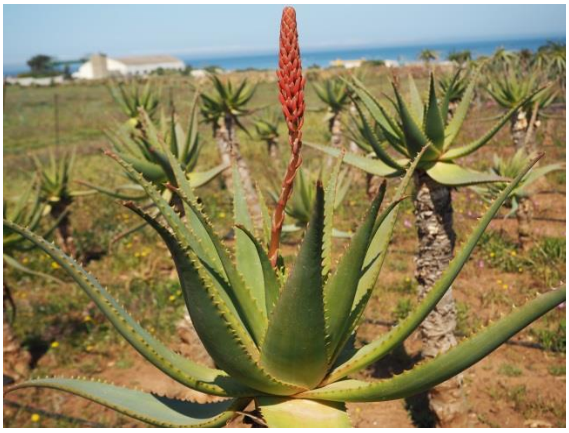

6.1. Can you identify this plant? [yes] [no]: if yes, give name of the plant ……………………………………………………………………………………… and continue, if no skip rest

6.2. Is it invasive or indigenous? ………………………………………………………………………………………………..

6.3. Have you personally seen this plant? [yes] [no] [not sure]

6.4. Is it [very common] [common] [moderate] [scarce] [very scarce] in your area?

6.5. Is it spreading and increasing in your area? [yes] [no] [same] [don't know]

6.6. Is this plant [beneficial] [harmful] [no impact] or [both harmful and beneficial]? Reason for answer

…….

………

6.7. Where did you learn about these benefits or impacts? [personal observation] [verbal communication] [school/university] [the media] [the internet] [other – specify]

……

……

6. Have you ever seen any articles with information about invasive alien species? [yes] [no], If yes, where? ....

7. Would you like to see invasive species removed and densities decreased in your area? [yes] [no] [unsure] [don't care] Reason: …….

8. Have you ever removed invasive species? [yes] [no] if yes, which ones and where? ………………………………………………………………………………………………

……………………………………………………………………………………………………
……………………………………………………………………………..

9. Do you know of any volunteer campaigns that manage/control IAS in your area? [yes] [no] if yes, which ones?
……………………………………………………………………………………………………
……………………………………………………………………………………………………
……………………………………………………………………………………….

10. Have you ever volunteered to manage IAS? [yes] [no], If no, would you like to join groups that manage/control IAS in your area? [yes] [no]

11. Have you heard of [iNaturalist/iSpot] [SAPIA] [City of Cape Town EDRR Face book page] [SANBI's Biological Invasion Directorate/Invasive species programme] [Working for Water] [Cape Nature] [SANParks]?

12. Have you ever reported the localities of invasive species? [yes] [no]: If yes in which platform do you report these localities to? [SAPIA] [iNaturalist] [City of Cape Town Face Book Page] [SANBI's Biological Invasion Directorate] [Working for Water] [SANParks] [Cape Nature] [report them to someone you know that works for these organisations] [you don't report them]. How many times in the past year you reported? [1-5][5-10][10-15][15-25][25-35][35-50] [more than 50 times] Name five species you reported
……………………………………………………………………………………………………
………………………………………………………………………………………..........................
....If no, would you be interested in learning how to report the localities of Invasive alien species? [yes] [no]. Reason:
……………………………………………………………………………………………………
………………..

13. Would you be happy to receive published information on Invasive alien species? [yes] [no] [only on certain species]. Reason
……………………………………………………………………………………………………
….

Demographic

1. Name: ……………………………………….
2. Gender [M][F]
3. Age ……………….
4. Ethnic group ……………………………….
5. Location ………………………………………………………….
6. Residency time in this location: [Born here] [> 20 years] [10-20 years] [5-10 years] [1-5 years] [<1 year]
7. Education level…………………………………….
8. Current or previous field of work
9. Contact details: ……………………………………………………………………………………….

Supplementary information file S2: A short description of the selected IAS: *Metrosiderous excelsa, Genista monspessulana, Pennisetum setaceum, Harmonia axyridis* and *Sus scrofa*.
Indigenous species: *Aloe arborescens*

**Species name**	**Native range**	**Growth form**	**Impact**
Metrosideros excelsa Sol. ex Gaertn	New Zealand	Tree	It is a problematic invader, displacing fynbos vegetation.
Genista monspessulana (L.) L.A.S.Johnson	Europe	Shrub	*G. monspessulana* forms dense monospecific stands, increases the fuel load for fires and displaces native species.
Pennisetum setaceum (Forssk.) Chiov.	North African arid Mediterranean area of the Atlas Mountains	Grass	*Pennisetum setaceum* is a highly aggressive pioneer and fire-adapted species that readily outcompetes native plants
Harmonia axyridis Pallas	eastern Asia	Invertebrate animal	*Harmonia axyridis* outcompete and displace native ladybird species,
Sus scrofa Linnaeus	Eurosia	Vertebrate animal	*Sus scrofa* destroy crops and pasture and habitat for indigenous plants and animals. They spread environmental weeds and could spread exotic diseases.
Aloe arborescens Mill.	South Africa	Shrub - tree	

Supplementary information S3 – Table S1: showing different linear models tried to assess if knowledge of *Metrosideros excelsa* is correlated with plant densities in different towns. The models are ordered by their relative AIC and the best selected one is highlighted in bold.

Model number	Model description	Log likelihoods	Number of parameters	delta.aics	Weight (wi)
**1**	**Metrosideros_ID ~ Residency + Education + Metrosideros_Density**	**-92.7919**	**12**	**0**	**0.323173**

2	Metrosideros_ID ~ Education + Metrosideros_Density	-97.121	8	0.657984	0.232571
3	Metrosideros_ID ~ Education	-98.2583	7	0.932913	0.202701
4	Metrosideros_ID ~ 1	-108.469	1	9.3548	0.003007
5	Metrosideros_ID ~ Residency	-104.513	5	9.441782	0.002879
6	Metrosideros_ID ~ Gender	-107.96	2	10.33703	0.00184
7	Metrosideros_ID ~ Metrocideros_Density	-108.397	2	11.21047	0.001189
8	Metrosideros_ID ~ Age	-106.229	7	16.87379	7.00E-05

Table S2: showing different linear models tried to assess if knowledge of *Pennisetum setaceum* is correlated with plant densities in different towns. The models are ordered by their relative AIC and the best selected one is highlighted in bold.

Model number	Model description	Log likelihoods	Number of parameters	delta.aics	Weight (wi)
**1**	**Pennisetum_ID ~ Education + Pennisetum_density + Age**	**-100.351**	**14**	**0**	**0.569819**
2	Pennisetum_ID ~ Residency + Education + Pennisetum_density	-96.8114	18	0.921027	0.359533
3	Pennisetum_ID ~ Age	-110.189	7	5.676259	0.033354
4	Pennisetum_ID ~ Education + Pennisetum_density	-109.316	8	5.93041	0.029374
5	Pennisetum_ID ~ Education	-111.833	7	8.964689	0.006443
6	Pennisetum_ID ~ 1	-120.169	1	13.63557	0.000623
7	Pennisetum_ID ~ Gender	-119.754	2	14.80664	0.000347
8	Pennisetum_ID ~ Residency	-116.985	5	15.26824	0.000276
9	Pennisetum_ID ~ Pennisetum_density	-120.166	2	15.63004	0.00023

Supplementary information S4 –: Summary of the parametric variables used in the analyses performed at different scales, their questions/explanation and variable type. The original wording and questions are given in a Supplementary material file 1.

Predictor Variable	Question/Explanation	Variable Type
Awareness of the term IAS	*"do you know what an invasive alien species is?: if yes, describe what IAS is"*	Categorical: yes/no<br>no= (person did not provide the correct definition);<br>yes = (person provided the correct definition)
Self-estimated knowledge of *Metrosideros excelsa*	Do you know this tree? (shown on a photograph)	Categorical yes/no<br>no= (person did not provide the correct species name);<br>yes = (person provided the correct species name)
Self-estimated knowledge of *Pennisetum setaceum*	Do you know this tree? (shown on a photograph)	Categorical: yes/no no= (person did not provide the correct species name); yes = (person provided the correct species name)
Alen plant density	How much people know in relation to the number of alien plant species in their towns	Continuous
Age	Respondent's age	Continuous: <20 years; 20-29 years;30-39 years;40-49 years;50-59 years;60-69 yeears;60-69 years;70-80 years
Gender	Respondents' gender	Categorical: Male/female
Education level	Whether respondents were educated or not	Categorical. Categories: Bachelor's degree, Honours degree, National Diploma, Matric, High School, Primary and not disclosed)

Residency	Respondent’s resident time in the areas	Categorical. Categories: Just visiting,<1year;1-5 year;5-10 years;10-20 years;>20 years

Supplementary information S5. Demographic details for the surveyed population from eight towns along the Berg River Catchment.

Town	Total respondents (n)	Male/Female (n)	Median age	Average residence time lowest/highest (yr)	Lowest/Highest Level of education
Gouda	39	19/20	20	1-5/>20	Grade 5/Grade 12
Hermon	40	11/29	37	1-5/>20	Grade 7/Grade 12
Morreesburg	39	12/27	39	1-5/>20	Grade 8/ Bachelor's Degree
Riebeek Kasteel	41	17/24	39	1-5/>20	Grade 12
Riebeek West	38	10/28	38	<1/>20	Grade 2/Grade 12
Tulbagh	30	19/11	35	<1/>20	Grade 4/ National Diploma
Wolseley	25	19/6	31	1-5/>20	Grade 6/ National Diploma
Hopefield	10	2/8	43	1-5/>20	High school/ National diploma
**Total of all respondents**	**262**				

Supplementary information S6: Significance levels of candidate models with categorical variables explaining correlation between knowledge of IAS (marked with * in Table S1).

What_is_invasive ~ Education

	Estimate	Std. Error	z	value	Pr(>|z|)
(Intercept)	1.099	1.155	0.951	0.34139	
Education: High school	-2.691	1.197	-2.248	0.0246	*
Education: Honours degree	13.467	882.744	0.015	0.98783	
Education: Matric	-2.291	1.187	-1.929	0.05371	.
Education: National diploma	1.204	1.56	0.772	0.44022	
Education: Primary	-4.682	1.537	-3.047	0.00231	**
Education: refused	-4.127	1.297	-3.182	0.00147	**

What_is_invasive ~ Location

	Estimate	Std. Error	z	value	Pr(>|z|)
(Intercept)	-2.48491	0.60093	-4.135	3.55E-05	***
Location: Hermon	-1.17865	1.1776	-1.001	0.31688	
Location: Hopefield	2.48491	0.87242	2.848	0.0044	**
Location: Moorreesburg	1.67398	0.69389	2.412	0.01585	*
Location: Riebeek-Kasteel	-16.0812	1018.665	-0.016	0.9874	
Location: Riebeek-West	0.02817	0.8503	0.033	0.97357	
Location: Tulbagh	2.21664	0.70488	3.145	0.00166	**
Location: Wolseley	2.07944	0.72648	2.862	0.00421	**

What_is_invasive ~ Age

	Estimate	Std. Error	z	value	Pr(>|z|)
(Intercept)	-2.2246	0.5263	-4.227	2.37E-05	***
Age: 18-29	0.8237	0.5918	1.392	0.164	
Age: 30-39	0.9561	0.6476	1.476	0.14	
Age: 40-49	0.9253	0.6468	1.431	0.153	
Age: 50-59	0.5382	0.7169	0.751	0.453	
Age: 60-69	0.3528	0.9241	0.382	0.703	
Age: >80	0.8383	1.2357	0.678	0.498	

What_is_invasive ~ Residency

	Estimate	Std. Error	z	value	Pr(>|z|)
(Intercept)	1.88E-14	1.00E+00	0	1.00E+00	
Residency >20	-1.64E+00	1.03E+00	-1.591	0.1115	
Residency: 1-5	-4.42E-01	1.09E+00	-0.406	0.6845	
Residency: 10-20	-2.21E+00	1.06E+00	-2.085	0.0371	*
Residency: 5-10	-8.65E-01	1.09E+00	-0.797	0.4254	

What_is_invasive ~ Gender + Residency + Age + Education + alien_density

	Estimate	Std. Error	z	value	Pr(>|z|)
(Intercept)	2.70E+00	2.13E+00	1.263	0.206491	
GenderMale	1.12E+00	4.32E-01	2.589	0.009613	**
Residency >20	-7.82E-01	1.33E+00	-0.589	0.556061	
Residency: 1-5	1.16E-01	1.42E+00	0.081	0.935316	
Residency: 10-20	-8.28E-01	1.41E+00	-0.586	0.558021	
Residency: 5-10	1.37E-02	1.41E+00	0.01	0.992224	
Age: 18-29	-3.51E-03	8.49E-01	-0.004	0.996699	
Age: 30-39	-1.88E-01	9.30E-01	-0.202	0.84007	
Age: 40-49	2.96E-01	9.68E-01	0.306	0.759801	
Age: 50-59	7.48E-01	1.05E+00	0.712	0.47664	
Age: 60-69	7.24E-01	1.20E+00	0.606	0.544742	
Age: >80	-1.53E+00	2.19E+00	-0.7	0.483695	
Education: High school	-2.59E+00	1.44E+00	-1.797	0.072261	.
Education: Honours degree	1.33E+01	1.46E+03	0.009	0.992698	
Education: Matric	-2.45E+00	1.45E+00	-1.687	0.091524	.
Education: National diploma	1.01E+00	1.81E+00	0.557	0.577474	
Education: Primary	-4.83E+00	1.80E+00	-2.683	0.007294	**
Education: refused	-3.82E+00	1.55E+00	-2.458	0.013974	*
alien_density	-9.31E-01	2.80E-01	-3.328	0.000876	***

What_is_invasive ~ Gender + Education

	Estimate	Std. Error	z	value	Pr(>ǀzǀ)
(Intercept)	0.5837	1.2002	0.486	0.62674	
GenderMale	1.2157	0.3945	3.082	0.00206	**
Education: High school	-2.792	1.2401	-2.251	0.02436	*
Education: Honours degree	12.7666	882.7442	0.014	0.98846	
EducationMatric	-2.2978	1.2279	-1.871	0.0613	.
Education: National diploma	1.2044	1.598	0.754	0.45101	
EducationPrimary	-4.7979	1.5732	-3.05	0.00229	**
Educationrefused	-4.3546	1.3416	-3.246	0.00117	**

What_is_invasive ~ Gender + Education + alien_density

	Estimate	Std. Error	z	value	Pr(>ǀzǀ)
(Intercept)	2.26E+00	1.44E+00	1.567	1.17E-01	
Gender: Male	1.1905	4.08E-01	2.92E+00	0.00355	**
Education: High school	-2.93E+00	1.39E+00	-2.118	0.0342	*
Education: Honours degree	1.25E+01	8.83E+02	0.014	0.98872	
Education: Matric	-2.6466	1.38E+00	-1.92E+00	0.05545	.
Education: National diploma	0.7788	1.7094	0.456	0.64869	
Education: Primary	-5.3206	1.7101	-3.111	0.00186	**
Education: refused	-4.1995	1.4892	-2.82	0.0048	**
alien_density	-0.8051	0.2509	-3.208	0.00134	**
